# A TP53-based immune prognostic model for muscle-invasive bladder cancer

**DOI:** 10.18632/aging.202150

**Published:** 2020-12-15

**Authors:** Hongyan Li, Huayi Lu, Wanli Cui, Yufan Huang, Xuefei Jin

**Affiliations:** 1 Jilin Key Laboratory of Urologic Oncology, Department of Urology, China-Japan Union Hospital of Jilin University, Changchun, China; 2 The First Affiliated Hospital of USTC, Division of Life Sciences and Medicine, University of Science and Technology of China, Hefei, Anhui, China

**Keywords:** MIBC, TP53, immune prognostic model, immune response, immune checkpoints

## Abstract

Background: Muscle-invasive bladder cancer (MIBC) patients are subject to unfavorable treatment options and a high recurrence rate. The status of TP53 mutations played an essential role in the progression and the prognosis of MIBC. The present study proposed to investigate the association between TP53 mutations and immunophenotype in MIBC.

Results: We established an immune prognostic model (IPM) ground on the immune-associated genes derived from variation analysis between wild-type TP53 and mutated TP53 TCGA-MIBC patients, and validated in another cohort from GEO database. Based on IPM, we divided MIBC patients into low and high risk subgroups. The high risk MIBC patients had higher proportions of macrophages M1, and lower proportions of T cells regulatory (Tregs) and activated dendritic cells than the low risk MIBC patients. Moreover, the expression of immune checkpoints genes (PD1, CTLA4, LAG3, HAVCR2 and TIGIT) was higher in the high risk patients than the low risk patients. In clinical application, IPM exhibited better survival prediction than conventional clinical characteristics.

Conclusions: Our investigation presented practical prognostic significance for MIBC patients and displayed the overarching landscape of the immune response in the MIBC microenvironment.

Methods: Data were obtained from The Cancer Genome Atlas (TCGA) and Gene Expression Omnibus (GEO). Differentially expressed genes (DEGs) analysis between the TP53 mutated and wild-type MIBC patients was conducted. The CIBERSORT algorithm was performed to evaluate the proportion of immune cell types. Gene expression profiles from the TCGA and GEO were used as training and testing cohorts to build and validate an immune-related prognostic model (IPM). Genes in the IPM model were first screened by univariate Cox analysis, then filtered by the least absolute shrinkage and selection operator (LASSO) Cox regression. A nomogram was finally established and evaluated by combining both the IPM and other clinical factors.

## INTRODUCTION

Bladder cancer is a prevalent malignant disease with 429,000 new cases and nearly 165,000 deaths worldwide annually [1, 2]. Bladder cancer develops along two different pathways: low phase non-muscle invasive bladder cancer (NMIBC) and high phase muscle-invasive bladder cancer (MIBC) [3]. Radiotherapy, combined with cisplatin-based chemotherapy, is the current standard method for high phase MIBC treatment. However, the recurrence rate of almost 70% leads MIBC patients to undergo long-term surveillance. Consequently, MIBC becomes more costly than other cancers from diagnosis to the end of life [3, 4]. Recent studies have shown that different types of tumor-infiltrating immune cells are involved in the development and prognosis of MIBC [5]. Immunotherapy has been studied as a new alternative for treating various types of cancer, especially those with an unfavorable prognosis by standard treatments [6]. Several immune-associated studies have been conducted to predict the prognosis of MIBC patients [5, 7, 8]. However, few studies have explored in detail the immune phenotype within the MIBC microenvironment and its relation with MIBC prognosis.

It has been said that no matter which orientation cancer investigation turns, TP53 gets into view. This is not only due to the essential significance in the inhibition of many human cancers but also for its leading role in various cancer-associated pathways, such as DNA repair, metabolism and antioxidant function [9]. In MIBC, patients with mutated TP53 get shorter overall survival than wild-type TP53 [10, 11]. Therefore, exploring the exact pathogenic mechanism of TP53 mutation status in MIBC and other cancers is crucial to obtain new therapeutic strategies and improve the prognosis. Remarkably, several recent studies have indicated that different immune responses are involved in the status of TP53 mutations [12, 13]. Although previous studies have found that TP53 mutations were associated with bladder cancer's clinical features, such as grade classification, cancer invasion, recurrence and poor prognosis [14]. However, their specific functions in the immune profiles of MIBC have not yet been fully elucidated. We performed a comprehensive analysis to explore the correlation between the TP53 mutation and the overall survival in the Cancer Genome Atlas (TCGA) MIBC cohort. Our results indicated that 15 immune-associated biological processes were inhibited in mutated TP53 MIBC patients. Furthermore, we identified four essential differentially expressed immune-associated genes that were posteriorly used to develop an immune prognostic model (IPM). We have shown that the proposed IPM can be employed as a useful prognostic strategy in patient management. The four identified genes can be used as potential therapeutic biomarkers for MIBC.

## RESULTS

### Relationship between immunotype and TP53 somatic mutations in MIBC

**Figure 1:**
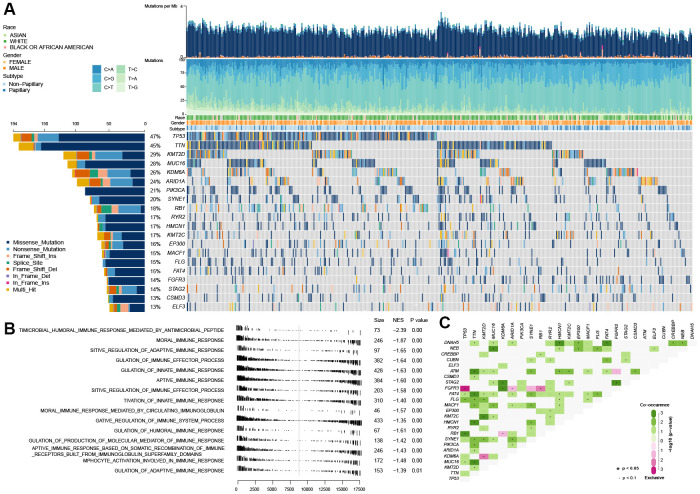
**Gene set enrichment analysis between the wild-type TP53 and mutated TP53 subgroup in TCGA MIBC dataset.** (**A**) The landscape of somatic mutations in TCGA MIBC dataset. (**B**) 13 inhibited immune-associated biological processes in mutated TP53 MIBC patients. (**C**) The landscape of somatic interactions of TP53 in TCGA MIBC dataset.

As shown in Figure 1A, TP53 mutation is the universal genetic mutation in MIBC. Although many studies have explored the function of TP53 mutations and found that TP53 was associate with some of the clinical features of bladder cancer, such as grade classification, cancer invasion, recurrence and poor prognosis [14], however, the particular function on immune profiles in MIBC has not been fully elucidated. In this study, we first explored the immune-associated biological processes in MIBC regarding TP53 status using gene expression matrix, somatic mutation data of 407 MIBC samples from the TCGA cohort, and matching clinical data. The patients with (n=194) and without (n=216) TP53 mutations were used to perform gene set enrichment analysis (GSEA). These results indicated that patients from the mutated TP53 group were remarkably enriched in 506 biological processes (BH-adjusted p < 0.05) (Supplementary Table 1). In addition, 15 immune-associated biological processes were fully inhibited in this group (Figure 1B). We also explored the somatic interactions of TP53 (Figure 1C) due to cancers being caused by simultaneous mutations of multiple genes. TP53 co-occurred with FAT4, FLG, and RB1 and were mutually exclusive with FGFR3 in the TCGA MIBC cohort.

### Identification of differentially expressed immune-associated genes in mutated and wild-type TP53 MIBC samples

To figure out the connections in TP53 mutation status with immune processes, we performed a differential expression gene analysis between wild-type and mutated TP53 groups of 1441 immune-associated genes acquired from the 15 immune-associated biological processes inhibited in the mutated TP53 group. 44 out of 1441 immune-associated genes were differentially expressed between wild-type and mutated TP53 MIBC samples (BH-adjusted p < 0.05 and absolute logFC > 1) (Supplementary Table 2).

### Development of an immune prognostic model (IPM) and assessment of its predictive ability in the TCGA MIBC cohort

To assess differentially expressed immune-associated genes’ predictive ability, we performed univariate cox regression of the expression matrix of these genes. We found 9 out of 44 immune-associated genes that were notably associated with overall survival (OS) (Supplementary Table 3). To get the most significant genes associated with prognostic worth, we used cox-proportional hazards analysis based on the L1-penalized LASSO estimation. We discovered four key genes (CTSG, TREML4, KRT1 and PPBP) that occurred more than 900 times in 1000 recurrences [15, 16]. The IPM score was established according to the four key genes expression weighted by the Cox regression coefficients: IPM risk score = (0.162 × CTSG expression) + (1.167 × TREML4 expression) + (0.164 × KRT1 expression) – (0.326 × PPBP expression).

**Figure 2:**
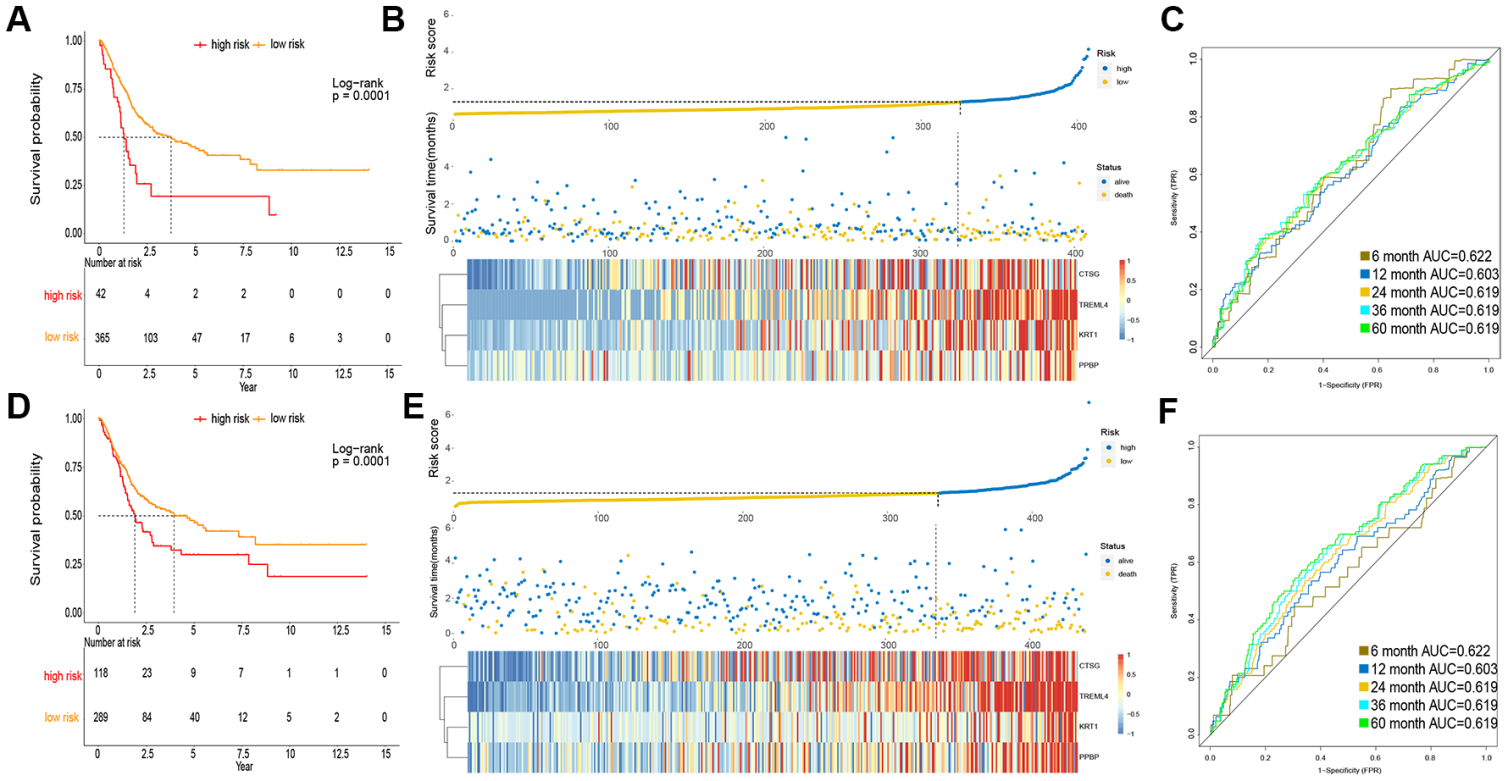
**Prognosis of IPM in TCGA MIBC cohort and meta-GEO MIBC cohort.** (**A**, **D**) Kaplan-Meier survival analysis in TCGA cohort and meta-GEO cohort, OS in low risk subgroup was higher than those in the high risk subgroup. (**B**, **E**) Risk assessment in TCGA cohort and meta-GEO cohort, the correlation between the risk score (upper) and the expression of four immune-associated genes (bottom). (**C**, **F**) Time-dependent ROC curves of IPM.

To obtain a rigid cutoff value to categorize the MIBC patients into high or low risk classes, we standardized four critical genes’ expression value with mean value = 0 and standard deviation = 1 [17]. We then used X-tile software to calculate the optimal cutoff point value (1.6) to classify patients into these two groups. In the TCGA cohort, the OS of patients in the high risk class was shorter than that of the low risk group (Figure 2A). Moreover, the high risk subgroup patients displayed a 2.30-fold higher risk (95% confidence interval (CI): 1.98–2.31, BH-adjusted p < 10^-3^) than the low risk subgroup patients. The risk score and expression distribution of the four essential genes are displayed in Figure 2B. The area under the ROC curve (AUC) was used to show the predictive ability of IPM (Figure 2C).

### Validation of the predictive ability of IPM in meta-GEO MIBC cohort

To verify the robustness of IPM constructed from the TCGA cohort, we assessed the metagene expression omnibus (meta-GEO) MIBC cohort, composed of 440 MIBC samples, with the same formula and cut off value used in the TCGA MIBC cohort. In the meta-GEO MIBC cohort, the OS of high risk group patients was lower than the low risk class (Figure 2D), as previously found in the TCGA MIBC cohort. The high risk patients displayed worse OS (HR: 2.61, 95% confidence interval (CI): 1.76–2.23, BH-adjusted p < 10^-3^) than the low risk patients, indicating the applicability of the developed IPM in different populations. The risk score and expression distribution of the four essential genes are displayed in Figure 2E. The IPM obtained from AUC of the meta-GEO MIBC cohort is shown in Figure 2F. Taken together, our analysis has shown that the developed IPM has proven to be robust when faced with different datasets and, therefore, can be used for further studies.

### Classification analysis of OS for IPM based on TP53 status in the TCGA MIBC cohort

**Figure 3:**
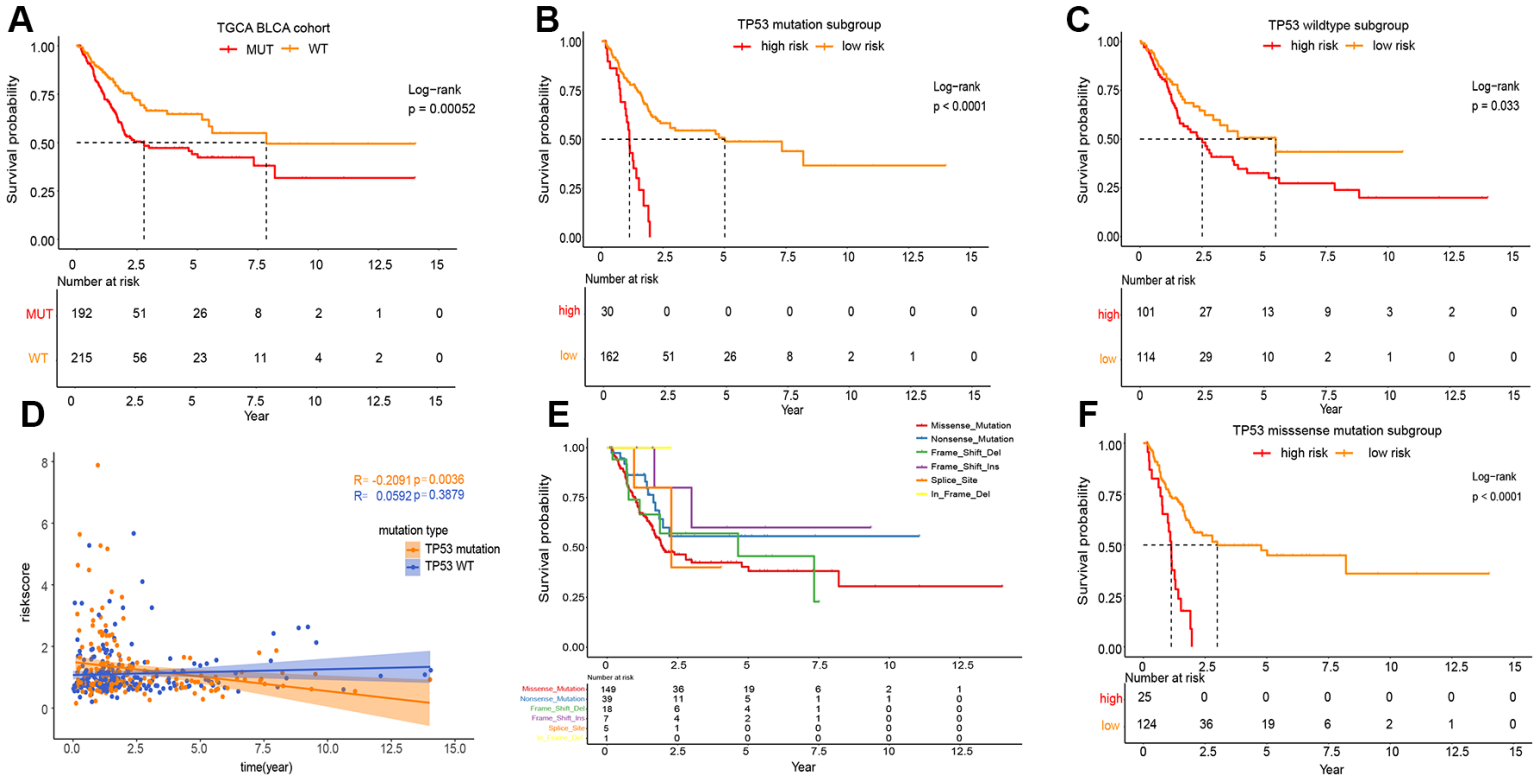
**Prognosis of different TP53 mutations in TCGA MIBC cohort.** (**A**) Kaplan-Meier survival analysis between the wild-type TP53 patients and mutated TP53 patients. (**B**) Kaplan-Meier survival analysis between the high and low risk subgroup in mutated TP53 MIBC patients. (**C**) Kaplan-Meier survival analysis between the high and low risk subgroup in wild-type TP53 MIBC patients. (**D**) Correlation analysis between risk score and survival time according to TP53 status. (**E**) Kaplan-Meier survival analysis among the different types of TP53mutations. (**F**) Kaplan-Meier survival analysis between the high and low risk subgroup in TP53 MIBC missense mutation patients.

As shown in Figure 3A, the phenotype of TP53 was meaningfully connected with the prognosis of patients with MIBC. To prove the relevance between the prognostic significance of IPM and TP53 status, we performed a classification analysis, separating the TCGA MIBC cohort into two groups based on TP53 status. Classification analysis demonstrated that IPM was significantly associated with OS in wild-type and mutated TP53 groups (Figure 3B, 3C). Moreover, correlation analyses showed that the risk score was significantly positively connected with OS in the wild-type TP53 group and negatively in the mutated TP53 group (Figure 3D). We performed classification analyses of several types of TP53 mutations, and we found that different TP53 mutations affect the prognosis of patients with MIBC (Figure 3E) [10, 18]. To prove the relevance between the prognostic significance of the IPM and TP53 mutations, we performed a predictive analysis of the TP53 missense mutation subgroup, which is the most common type of TP53 mutation. As expected, the TP53 missense mutation was divided into high and low risk groups by IPM (Figure 3F).

### Immune landscape comparison between the low and high risk MIBC patients

**Figure 4:**
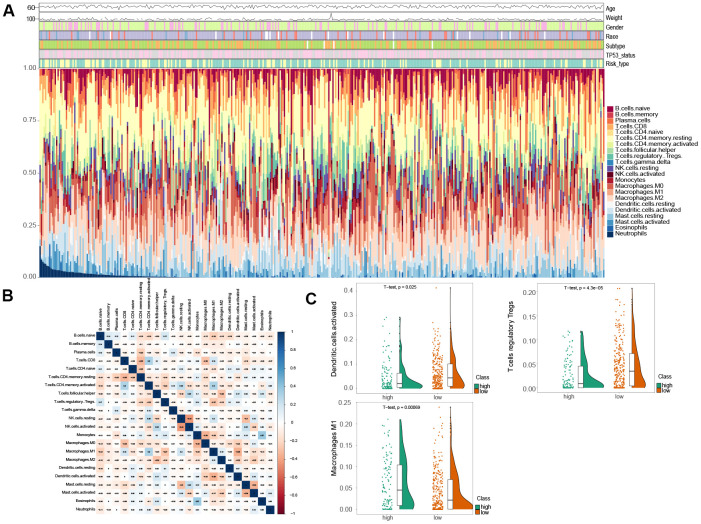
**Comparison of immune infiltration landscape between the high and low risk MIBC patients.** (**A**) Immune cell proportions between the high and low risk MIBC patients. (**B**) Correlation matrix of 22 types of immune cell proportions. (**C**) Significantly difference of immune cells between the high and low risk MIBC patients.

To assess the variation of 22 immune infiltrated cell types between low and high risk MIBC patients, we performed an immune infiltration analysis using CIBERSORT analytical tool with the LM22 signature matrix [19]. The immune landscape of the 407 TCGA MIBC cohort was summarized by representing the proportions of the 22 immune cell types within and between low and high risk groups (Figure 4A). The proportions of diverse subtypes of tumor-infiltrating immune cells were slightly moderately correlated (Figure 4B). The high risk MIBC patients had significantly higher proportions of macrophages M1, while expressively lower proportions of T cells regulatory (Tregs) and activated dendritic cells than the low risk MIBC patients (BH-adjusted p < 0.05) (Figure 4C). Hence, our results indicated that aberrant immune cell infiltration and the heterogeneity of tumor infiltration in MIBC might serve as prognostic indicators and targets for immunotherapy and have significant clinical implications.

**Figure 5:**
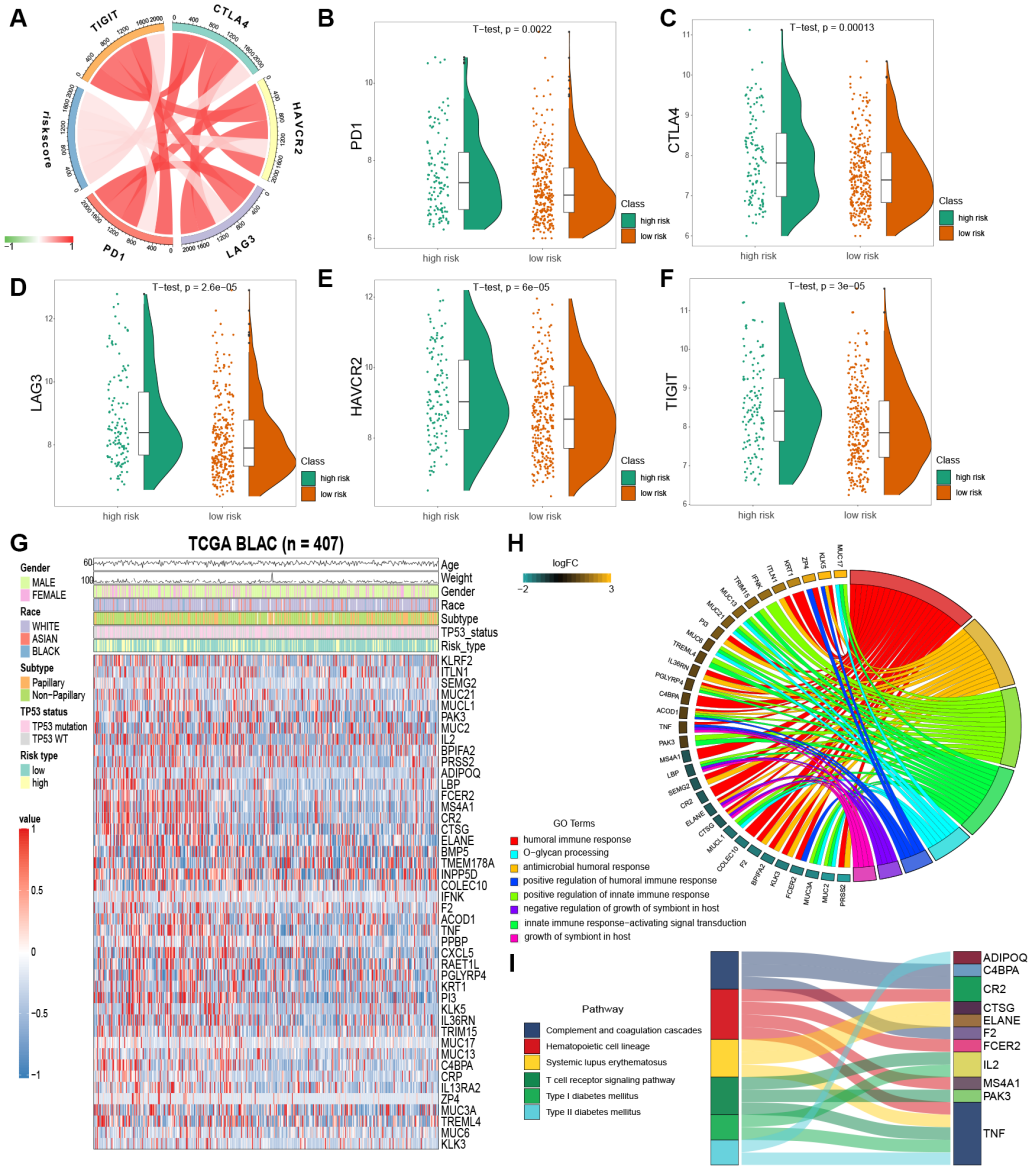
**Enrichment analysis of IPM.** (**A**) Correlation between the risk score and the expression level of immune checkpoints genes. (**B**–**F**) The expression level of immune checkpoints genes in the high and low risk MIBC patients. (**G**) Heatmap plot of immune-associated genes that were differentially expressed between the high and low risk MIBC patients. (**H**) Enrichment of biological processes for immune-associated genes that are shown in the circular plot. (**I**) Enrichment of KEGG pathways for immune-associated genes that are shown in the sankey plot.

Immune checkpoint inhibition has been a way explored by many novel anti-tumor agents, which have been used successfully in different types of cancer [20]. In bladder cancer, drugs targeting immune checkpoints genes have also been indicated to play anti-cancer function by reversing tumor immunosuppressive effects [21]. Therefore, we estimated the correlation between the risk score of MIBC patients and expression level of vital immune checkpoints genes (PD1, CTLA4, LAG3, HAVCR2 and TIGIT) (Supplementary Table 4). We demonstrated that the risk score of MIBC patients was significantly associated with the expression of PD1, CTLA4, LAG3, HAVCR2 and TIGIT (BH-adjusted p < 0.05) (Figure 5A). Furthermore, we showed that the expression level of PD1, CTLA4, LAG3, HAVCR2 and TIGIT genes in the high risk MIBC subgroup was significantly higher than in the low risk subgroup (BH-adjusted p < 0.01) (Figure 5B–5F). These data suggest that the bleak prognosis of high risk MIBC patients may be partially due to the immunosuppressive microenvironment.

### Variation of biological processes and pathways in low and high risk MIBC subgroup patients

We filtered the 44 immune-associated genes differentially expressed between low and high risk subgroups of MIBC patients by risk score association using the correlation analysis method. This analysis identified 25 immune-associated genes as risk score associated (Pearson correlation coefficient >0.2 and BH-adjusted p < 0.05; Figure 5G). GO and KEGG analyses were used to establish the inherent biological functions and pathways involved in these genes (FDR < 0.0001 and FDR < 0.001, respectively; Figures 5H, 5I) (Supplementary Tables 5 and 6). The 25 immune-associated genes related to the risk score in the TCGA MIBC cohort were principally enriched in the humoral and innate immune responses and T cell receptor signaling pathway (Figures 5H, 5I).

### IPM exhibits a better survival prediction than conventional clinical characteristics

**Figure 6:**
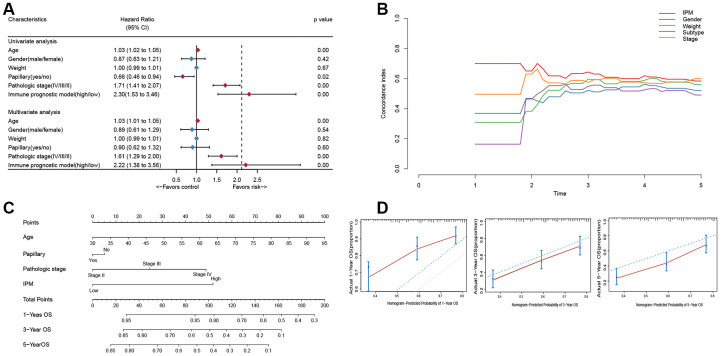
**The connection between IPM and conventional clinical characteristics.** (**A**) Univariate and multivariate regression analysis of IPM and clinical characteristics in prognostic value. Blue displays no statistical significance, and red displays statistical significance. (**B**) Comparison of c-index among different clinical characteristics. (**C**) Nomogram for predicting the probability of 1, 3, and 5 years OS for MIBC patients. (**D**) Calibration plot of the nomogram for predicting the probability of OS at 1, 3, and 5 years (red lines); dash lines indicate the actual probability. The vertical mark lines in the top x-axis (marginal rugs) stand for the distribution of samples in each fitting curve.

To compare the prognostic value of IPM with conventional clinical characteristics in the TCGA MIBC cohort, we performed univariate and multivariate Cox regression analyses involving five conventional clinical characteristics (age, gender, weight, subtype, and pathological stage). The univariate Cox regression analysis showed that IPM was independent of the prognostic aspect, accordingly proving its robustness to predict MIBC prognosis independently (Figure 6A). Furthermore, the multivariate Cox regression analysis demonstrated that IPM was meaningly associated with the survival time (BH-adjusted p < 0.01) and the highest median risk score (HR = 2.22, 95% CI = 1.38 – 3.56). In addition to the Cox regression analysis, we also estimated the c-index between IPM and conventional clinical characteristics. The c-index value of IPM (0.7181) was higher than other conventional clinical characteristics (0.1821 – 0.6438) (Figure 6B). Taken together, these results demonstrated that IPM has a better survival prediction than conventional clinical characteristics.

### Construction and validation of the individualized nomogram on IPM

To predict the prognosis of MIBC patients for clinicians, we used independently predictors (age, subtype, pathologic stage and IPM) that were identified in the multivariate Cox regression analysis (BH-adjusted p < 0.05) to construct the nomogram. As shown in Figure 6C, the IPM presented more risk points than other predictors. The c-index of the nomogram was 0.72 (95%CI, 0.61 to 0.86) using 1000 bootstrap replicates. The calibration curve displayed good agreement between the prediction and the investigation (Figure 6D). Except for the probability of 1-year survival, others were significantly lower than that of the nomogram (BH-adjusted p <BH-adjusted p < 0.01).

## DISCUSSION

TP53 mutations are related to high grade, invasive tumor, low recurrence and adverse clinical outcomes in bladder cancer [14]. Recent studies have found that TP53 mutation can increase the gene expression involved in immune checkpoints, activate T-effector cells, and elevate interferon-γ in lung adenocarcinoma [12]. Moreover, co-occurring TP53/KRAS mutation displayed more benefit from PD-1 inhibitors [12]. TP53 also was reported to be a predictor in immunotherapy of PD-1 in lung carcinomatosis [22]. Regarding bladder cancer, it has been demonstrated that anti-PD-1 antibodies enhance radiotherapy-induced anti-tumor immunity. However, the molecular mechanism of TP53 mutations in MIBC immunophenotype regulation and MIBC's prognosis is unknown. Therefore, the immune-associated effects of TP53 mutations in bladder cancer must be studied.

Furthermore, the development of advanced immune-associated prognostic models would be useful to identify biomarkers, evaluate the immune state of MIBC patients, and classify them to enhance the effectiveness of immunotherapy. In recent years, tumor immune signatures have been established and identified in various cancers [23–25]. Even though some studies have attempted to illustrate immune signatures in bladder cancer [26, 27], the essence of the local immune landscape in MIBC prognosis and prediction has not been fully elucidated. In the present study, we explored the character of TP53 mutations in the modulation of immune signature in MIBC. The GSEA analysis showed that the mutated TP53 group had a significantly lower immune phenotype than wild-type TP53. Moreover, differential expression analysis of these immune-associated genes showed that 15 immune-associated biological processes were all inhibited in the mutated TP53 group. Subsequently, a cox-proportional hazards analysis based on the L1-penalized LASSO estimation pointed four key genes (CTSG, TREML4, KRT1 and PPBP) used to construct a novel immune prognostic model (IPM) to predict the prognosis of MIBC patients.

These four key genes are involved in various cancer-related immune processes. Cathepsin G (CTSG), an azurophil granule protease, can lead to breast cancer cell migration [28, 29] and can enter tumor endosomes by binding to a cell surface receptor [30]. Previous studies have shown that CTSG degraded MHC I on the human glioblastoma cell surface of primary immune cells. CTSG activity has been presented as a new way to glioblastoma treatment [31]. Besides, high CTSG expression was correlated with poor outcomes in treating acute myeloid leukemia (AML) [32]. The triggering receptor expressed on myeloid cells 4 (TREML4) is a Ig superfamily member and rarely reported in cancer [33]. However, we believe that TREML4 could be a new immunotherapeutic target because a TREML4 deficiency causes a fail in INF-I production by macrophages due to a decrease of phosphorylation level in the signal transducer and activator of transcription 1 (STAT1). Moreover, previous studies have found that TREM1 dramatically promotes proliferation and invasion in Hepatocellular Carcinoma (HCC) and TREM1 expression has been associated with poor survival in HCC patients [34]. In addition, high TREM2 expression was inversely associated with unfavorable prognosis in gastric cancer and, therefore, could be a useful prognostic biomarker [35]. Keratin 1 (KRT1) is a member of the keratin family, which is involved in protein binding [36], carbohydrate-binding [37] and structural constituent of the epidermis [38]. Recently, it was found that KRT1 was highly expressed in breast cancer cells, and, thus, pointed as a new marker for breast cancer targeting [39]. In addition, KRT1 was significantly overexpressed in colon cancer, especially in its later phase [39]. Finally, pro-platelet basic protein (PPBP), also known as CXCL7, is a biomarker of several cancer types, such as renal cell cancer [40], lung cancer [41] and colorectal cancer [42]. PPBP has been reported to be up-regulated in the peripheral blood of early-stage renal cell carcinoma patients [40]. PPBP accelerated the development of renal cell carcinoma by promoting cell proliferation [43]. In colorectal cancer, the expression of PPBP was significantly correlated with sex, TNM and T stages [42].

We demonstrated by different approaches that our IPM exhibited better survival prediction than traditional clinical features. Subsequently, we performed a comprehensive evaluation that integrated the IPM with conventional clinical characteristics (age, gender, weight, subtype, and pathological stage). The calibration curve displayed good agreement between the prediction and the clinical characteristics evaluated for 1, 3 and 5 years OS. Our main superiority is that it presents a complementary perspective on individual tumors and establishes a unique scoring method for MIBC patients. Therefore, our nomogram could be used as a promising tool for clinicians in the future.

The stage of elimination is an upgraded model of cancer immunosurveillance. The innate and adaptive immune systems co-operate to find the existence of the progression of tumors and devastate it before it becomes worse [44]. Like Type I IFNs, some danger signals can induce the growth of tumors, motivate dendritic cells and promote the adaptive immune responses [45]. In the equilibrium stage, the adaptive immune system restrains tumor cell outgrowth and forms the tumor cells’ immunogenicity. IL-12, IFN-γ, CD4^+^ and CD8^+^ T cells are responsible for retaining the occult tumor cells [44, 46].

The cancer immunoediting process involves three stages: elimination, equilibrium and escape [44]. In the escape stage, tumor cells that have passed through the former two steps and have obtained the ability to escape from immune recognition become visible and progressively growing tumors. Therefore, the occurrence of tumor escape is based on the establishment of an immunosuppressive status within the tumor microenvironment [44, 47]. Regulatory T cells (Tregs) are one of the main types of immunosuppressive cells responsible for restraining host-protective anti-tumor responses. Activated Tregs can express PD1, PDL1 and CTLA4 to inhibit tumor-specific T lymphocytes functions [44, 48]. We compared the immune infiltration of 22 immune cell types between low and high risk MIBC patients to explore the immune mechanisms and evaluate the reach of the proposed IPM as cancer immunotherapy. The results showed that the high risk MIBC patients had more macrophages. The patients with (n=194) and without (n=216) TP53 mutations, and fewer Tregs and activated dendritic cells than the low risk MIBC patients. Furthermore, the gene expression levels of PD1, CTLA4, LAG3, HAVCR2 and TIGIT in the high risk MIBC subgroup were significantly higher than those in the low risk subgroup. Therefore, the risk score obtained from IPM was consistent with the ability of immune infiltration to decide the expressed value of immune checkpoint genes. This suggests that the inferior prognosis of the high risk patients may have resulted from a more excellent immunosuppressive environment and an increased expression level of immune checkpoint genes. Consequently, our results also suggest that immune checkpoint gene inhibitors should be potentially more effective in high risk MIBC patients, which would result in a considerable improvement in prognosis.

## CONCLUSION

The appointments presented here can present some limitations due to being based on retrospective data and, therefore, they should be further validated by prospective studies. Furthermore, the four key immune-associated genes used to construct the IPM should also be detected in experimental studies to ensure their clinical application. On the other hand, the present work provides new insights into the MIBC immune microenvironment and immune-associated therapies. For the first time, it was proposed an IPM based on TP53 mutations and four immune-associated genes. The proposed IPM presented a significantly effective prognostic for MIBC patients and illustrated an overarching landscape of immune response in the MIBC microenvironment. Remarkably, the development and validation of the IPM presented an immunological perspective to elucidate the mechanisms in the clinical outcomes of MIBC and potentially could be used as a reference for the study of other types of cancer.

## MATERIALS AND METHODS

### Cancer Genome Atlas (TCGA) data acquisition and processing

The gene expression matrix, somatic mutation data of 407 MIBC samples and their matching clinical features were acquired from the Cancer Genome Atlas (TCGA) website (https://portal.gdc.cancer.gov/repository). RNA-seq count data were downloaded using Illumina HiSeq platforms and annotated as Homo_sapiens. GRCh38.91.chr.gtf file (http://asia.ensembl.org/index.html). The function of variances stabilizing transformation (VST) normalization method of the DESeq2 package in R software was used to normalize the expression data [49]. The mean gene expression level was employed when multiple gene symbols were located. Moreover, we removed the genes that sum gene expression value to be less than 100 to filter out the low abundance data.

### Gene Expression Omnibus (GEO) data acquisition and processing

The four gene expression matrix of microarray data from GSE13507 based on platform GPL6102 (257 samples used in this study), GSE32549 based on platform GPL6947 (132 samples used in this study), GSE48075 based on platform GPL6947 (143 samples used in this study), and GSE48276 based on platform GPL14951 (117 samples used in this study) were obtained from the Gene Expression Omnibus (GEO) database (https://www.ncbi.nlm.nih.gov/geo/). The mean gene expression level was employed when multiple gene symbols were located. Genes were removed when its sum gene expression value was less than 100 to filter out the low abundance data. Moreover, the clinical data of four datasets with survival details were combined into the meta-GEO MIBC cohort (n=440) from 649 GEO patients to confirm the immune prognostic model. Combat method in sva R package was applied to remove the batch effects [50, 51].

### Gene Set Enrichment Analysis (GSEA)

We divided the TCGA cohort into two groups, wild-type TP53 (n = 216) and mutated TP53 (n = 194), to show the variations in immunological pathways and related genes using GSEA methods [52]. The MSigDB gene sets file (c5.bp.v7.0.symbols.gmt) was chosen as the reference gene set with permutations of 10^4^. FDR < 0.05 was appointed as the threshold.

### Differentially expressed genes (DEGs) and functional analysis

The R package DESeq2 was used to filter out the differentially expressed genes (DEGs) between the wild-type and mutated TP53 groups. Absolute log_2 _(fold change) > 2 and adjusted p values < 0.01 were set as the cut-off criteria to indicate significant statistically difference [49]. Gene Ontology (GO) annotation and Kyoto Encyclopedia of Genes and Genomes (KEGG) pathways analyses were performed using the clusterProfiler R package to explore the significant biological processes, highlight the pathways associated with DEGs, and assess the biological implications of the prognostic model [53]. The notable biological processes and pathways were presented using GOplot [54] and ggalluvial (version 0.10.0. https://CRAN.R-project.org/package=ggalluvial) R packages, respectively.

### Immune prognostic model (IPM) development and validation

To predict the prognosis of patients with MIBC, we constructed an immune prognostic model (IPM) as previously described [55]. We integrated 407 MIBC samples expression profiles, mutation data and survival information from TCGA MIBC samples to subsequent analyses. The expression matrix of the DEGs in immune-associated genes sets of GSEA from 407 MIBC was analyzed using univariate cox regression analysis. The significant genes with BH-adjusted p value < 0.01 were subject to the least absolute shrinkage and selection operator (LASSO) analysis with L1-penalty. LASSO is a widely used method for dealing with the very high dimensional space of predictors such as gene expression profiles [56]. Thus, the critical immune-associated genes, significant in univariate cox regression analysis, were screened out by the LASSO method. Finally, a comparatively small portion of non-zero weight genes remained. Most of the potential indicators were reduced to zero. Hence, we decreased the number of immune genes using LASSO-penalized cox regression. In this study, we performed LASSO-cox analysis using glmnet R package, and picked up immune-associated genes that appeared more than 900 times in 1000 repetitions [15, 57]. X-tile 3.6.1 software (Yale University, New Haven, CT, USA) was employed to define the best cutoff for categorizing low or high risk MIBC patients. The predictive ability of the IPM was assessed by the log-rank test and Kaplan-Meier survival analysis.

### Estimation of immune cell type fractions

CIBERSORT analytical tool [19] was employed to quantify the immune cell distributions in wild-type TP53 and mutated TP53 MIBC. The LM22 gene signature was designed to evaluate the possibility of leukocyte deconvolution from bulk tumors. LM22 signature matrix file contains 547 genes and enables highly sensitive and particular distinction of 22 human hematopoietic cell phenotypes, including T cell subtypes, B cell subtypes, NK cell subtypes, plasma cells subtypes and myeloid subtypes. For every sample, the sum of all estimates of 22 immune cell subtype fractions was equivalent to 1.

### Comparison between IPM and traditional clinical characteristics

We performed univariate and multivariate cox regression analyses for MIBC patients to compare the predictive ability of IPM and traditional clinical characteristics. The traditional clinical characteristics used here were obtained from survival information of 407 MIBC patients, including age, gender, weight, pathological stage and diagnosis type.

### Assessment of nomogram performance

The nomogram was used to show the predicted probability of survival for 1 year, 3 and 5 years, based on the outcome of multivariate analysis. The calibration curve and concordance index (c-index), which were generated by the rms (Version: 5.1–3.1; https://CRAN.R-project.org/package=rms) R package [58], decided the predictive exactness and discriminative capacity of a nomogram. The calibration curve indicated the distinction between the actual overall survival rate and the nomogram's predicted probability. A calibration curve closer to the diagonal dotted line suggests a better predictive effect. The c-index is mainly used to assess the predictive power of the model. The model impact can be equivalent to the area under the ROC curve (AUC). We calculated c-index by a bootstrap approach with resampling 1000 times [59]. For the statistical tests analyzed in this study, BH-adjusted p < 0.05 was used as a threshold to indicate statistically significant differences.

## Supplementary Material

Supplementary Table 1

Supplementary Tables 2, 3 and 4

Supplementary Table 5

Supplementary Table 6
