# Supplementary material for: A TP53-based immune prognostic model for muscle-invasive bladder cancer
Source: Aging (Albany NY). 2020 Dec 15;13(2):1929–46. doi: 10.18632/aging.202150 (PMC7880361; doi:10.18632/aging.202150)
Supplement: Supplementary Tables 2, 3 and 4 [file aging-13-202150-s002.pdf]

SUPPLEMENTARY TABLES

Supplementary Table 2. Differentially expressed immune-related genes between TP53 WT and TP53 MUT MIBCs.

Gene	BaseMean	Log2FoldChange	lfcSE	Stat	P value	Padj
INPP5D	1854.711104	-1.331192711	0.109912	-12.1115	9.19E-34	5.73E-30
MUC17	6.785766424	3.423430873	0.403068	8.493431	2.01E-17	1.39E-14
COLEC10	16.41223701	-1.518229034	0.19155	-7.92602	2.26E-15	1.09E-12
MUC3A	386.6954841	-1.666152055	0.222744	-7.48011	7.43E-14	2.32E-11
KRT1	666.6288899	2.256194309	0.315835	7.143583	9.09E-13	1.87E-10
IL13RA2	114.8086152	1.381038267	0.199597	6.919142	4.54E-12	6.69E-10
KLK5	639.2089362	3.012235524	0.447785	6.726965	1.73E-11	2.00E-09
ITLN1	314.1628376	2.063811109	0.322884	6.391796	1.64E-10	1.30E-08
MUC13	39.3318094	1.689564373	0.26715	6.3244	2.54E-10	1.88E-08
TNF	74.05026623	1.076075988	0.173247	6.21122	5.26E-10	3.37E-08
TRIM15	14.85020364	1.796347117	0.291317	6.1663	6.99E-10	4.25E-08
MUC2	1073.476454	-1.872285676	0.308671	-6.06564	1.31E-09	6.97E-08
FCER2	41.01428292	-1.629011171	0.270628	-6.01938	1.75E-09	8.67E-08
F2	18.10754493	-1.539812425	0.264972	-5.81122	6.20E-09	2.50E-07
TMEM178A	127.5392241	-1.005751905	0.173451	-5.79849	6.69E-09	2.68E-07
PRSS2	294.2979381	-1.951340192	0.33999	-5.73941	9.50E-09	3.60E-07
MUCL1	47.38242697	-1.433814436	0.254758	-5.62814	1.82E-08	6.15E-07
MUC6	27.50267307	1.281605722	0.232651	5.508708	3.61E-08	1.11E-06
BMP5	146.0012169	-1.313917978	0.251061	-5.23347	1.66E-07	3.96E-06
CXCL5	492.5128235	1.447470474	0.277447	5.217111	1.82E-07	4.28E-06
MUC21	48.10028086	1.57242524	0.304889	5.157366	2.50E-07	5.67E-06
CRP	7.047758192	1.768788946	0.350343	5.048736	4.45E-07	9.10E-06
ELANE	5.561058025	-1.156174197	0.234959	-4.92074	8.62E-07	1.56E-05
CTSG	116.8657596	-1.173494097	0.240986	-4.86956	1.12E-06	1.95E-05
RAET1L	65.33216009	1.125183807	0.236677	4.754085	1.99E-06	3.10E-05
PI3	6530.207297	1.496468891	0.314881	4.752485	2.01E-06	3.11E-05
PAK3	51.24237471	1.028488952	0.22018	4.67113	3.00E-06	4.27E-05
BPIFA2	9.044188556	-1.552672656	0.341593	-4.54539	5.48E-06	7.01E-05
IL36RN	208.5455292	1.195186363	0.265789	4.496741	6.90E-06	8.43E-05
TREML4	3.035898091	1.196997555	0.266379	4.493596	7.00E-06	8.55E-05
LBP	56.44304651	-1.060024831	0.238228	-4.44962	8.60E-06	0.0001015
PGLYRP4	158.3369183	1.131984695	0.255716	4.42672	9.57E-06	0.0001109
ZP4	1.384827197	2.740193555	0.675698	4.055351	5.01E-05	0.000433
C4BPA	11.83703756	1.11824931	0.281722	3.96933	7.21E-05	0.0005837
ADIPOQ	41.22982002	-1.880092603	0.482849	-3.89375	9.87E-05	0.0007568
MS4A1	199.7270789	-1.058261639	0.272528	-3.88314	0.00010312	0.0007833
IFNK	4.438731702	1.844122551	0.475738	3.876338	0.00010604	0.0008006

PPBP	16.3034348	1.010430324	0.262607	3.847685	0.00011924	0.0008795
CR2	180.5723803	-1.145738436	0.298234	-3.84174	0.00012216	0.0008956
ACOD1	3.209527907	1.098973015	0.293156	3.748769	0.00017771	0.0012264
IL2	1.674166645	-1.037857916	0.302595	-3.42986	0.00060389	0.0033249
KLK3	31.97130664	-1.573014493	0.480084	-3.27654	0.00105086	0.0052178
KLRF2	2.275614677	-1.023321575	0.317905	-3.21895	0.00128661	0.0061484
SEMG2	5.609722123	-1.119485718	0.359992	-3.10975	0.00187245	0.0083153

Supplementary Table 3. Univariate Cox regression analysis of differentially expressed immune-related genes.

Gene	HR	Z	Pvalue	Lower	Upper
BPIFA2	0.4793007	-3.19996146	0.00137446	0.30547927	0.75202865
KRT1	1.0945601	3.04094672	0.002358356	1.03263949	1.16019369
CTSG	1.1596203	2.56126922	0.01042905	1.03537893	1.29877008
TREML4	2.0823697	2.51592625	0.011872002	1.17596396	3.68741213
ELANE	1.7211129	2.51353421	0.011952818	1.12702529	2.6283612
MUC2	0.9028762	-2.36875478	0.017848083	0.82968624	0.98252244
TMEM178A	0.8130005	-2.15627734	0.031062015	0.67354587	0.98132852
PPBP	1.2036616	2.0668929	0.038744246	1.009636	1.43497394
RAET1L	1.157798	2.04852701	0.040508388	1.00635453	1.33203172
F2	0.7377252	-1.95931492	0.050075918	0.54418364	1.00010077
ADIPOQ	1.1276578	1.94118714	0.052235583	0.99883855	1.27309078
ITLN1	0.9045497	-1.90481201	0.056804512	0.81583703	1.00290883
PI3	1.0391878	1.67985532	0.092985468	0.99361088	1.08685542
C4BPA	0.791979	-1.64174983	0.10064186	0.59950851	1.04624152
INPP5D	0.9110958	-1.53367692	0.125109154	0.80888886	1.02621703
IFNK	0.7252585	-1.51608925	0.129496787	0.47878587	1.09861207
SEMG2	0.7812814	-1.49614551	0.134615741	0.56543718	1.07951985
IL36RN	1.077922	1.49606391	0.134637001	0.97700168	1.1892669
KLK5	1.0503211	1.4414847	0.149447798	0.98249599	1.12282836
TNF	0.8800157	-1.36851191	0.171151896	0.73280819	1.05679436
PGLYRP4	1.0777881	1.29292472	0.196037095	0.96208966	1.20740004
ACOD1	0.6651285	-1.26478914	0.205946925	0.35356848	1.25123139
CRP	0.8223409	-1.24966551	0.211421761	0.60509001	1.11759319
MUC21	1.0778289	1.20902356	0.226653794	0.95451528	1.21707329
ZP4	1.2053951	1.03709029	0.29969378	0.84684805	1.71574751
CR2	1.0445565	1.00597241	0.314428844	0.95950299	1.13714938
KLRF2	0.7381797	-0.86563715	0.386689174	0.37124151	1.46780272
TRIM15	0.8997109	-0.80566634	0.42043526	0.69574183	1.16347699
CXCL5	1.0324815	0.69564429	0.486651619	0.94355936	1.12978379
LBP	0.9502898	-0.67822945	0.49762622	0.82009479	1.10115414
MUC17	0.9070841	-0.6713751	0.501981605	0.68234945	1.205836

MUC3A	0.9750718	-0.50184944	0.615773435	0.88352562	1.07610343
PRSS2	0.9800865	-0.5000856	0.617014804	0.90578991	1.0604773
BMP5	0.9686302	-0.4442206	0.656883099	0.84156037	1.1148868
COLEC10	0.9146062	-0.441049	0.659177525	0.61512553	1.35989237
MUC6	1.0271493	0.31456764	0.753089952	0.86925826	1.21371937
PAK3	1.0261812	0.28366326	0.776668449	0.85836436	1.22680759
MUCL1	1.0185821	0.26652602	0.789834121	0.88960086	1.16626406
FCER2	1.0147179	0.20229408	0.839686831	0.88078	1.16902341
MUC13	0.9879315	-0.17040276	0.864693405	0.85916229	1.13600037
IL2	1.0557711	0.13710304	0.890949347	0.48598951	2.29357335
MS4A1	0.9936317	-0.13361744	0.893705091	0.90474645	1.09124934
IL13RA2	1.0082233	0.13085062	0.895893478	0.89182927	1.13980812
KLK3	1.0003818	0.00411676	0.996715308	0.83412129	1.19978215

Supplementary Table 4. Analysis of correlations between risk score and immune checkpoints.

Variable 1	Variable 2	Pearson correlation coefficient	P value
CTLA4	PD1	0.888288098	6.21E-139
CTLA4	TIGIT	0.886304303	1.78E-137
CTLA4	HAVCR2	0.831323626	2.32E-105
CTLA4	LAG3	0.844513955	6.75E-112
CTLA4	riskscore	0.186199337	0.000158114
HAVCR2	PD1	0.834320086	8.55E-107
HAVCR2	TIGIT	0.811648076	1.35E-96
HAVCR2	LAG3	0.839543509	2.30E-109
HAVCR2	riskscore	0.179520696	0.000272458
LAG3	PD1	0.87450565	2.44E-129
LAG3	TIGIT	0.804948762	7.60E-94
LAG3	riskscore	0.172904677	0.000458466
PD1	TIGIT	0.887113217	4.56E-138
PD1	riskscore	0.142612516	0.003939071
riskscore	TIGIT	0.201245306	4.32E-05
